# Medicinal Plants Used in the Ejisu-Juaben Municipality, Southern Ghana: An Ethnobotanical Study

**DOI:** 10.3390/medicines6010001

**Published:** 2018-12-20

**Authors:** Kwame Sarpong Appiah, Clement Peprah Oppong, Hossein Korrani Mardani, Richard Ansong Omari, Sylvia Kpabitey, Christiana Adukwei Amoatey, Siaw Onwona-Agyeman, Yosei Oikawa, Keisuke Katsura, Yoshiharu Fujii

**Affiliations:** 1 United Graduate School, Tokyo University of Agriculture and Technology, 3-5-8 Saiwaicho, Fuchu, Tokyo 183-8509, Japan; ksappiah90@gmail.com (K.S.A.); hmardani26@yahoo.com (H.K.M.); talk2jafakingonline@gmail.com (R.A.O.); kkatsura@go.tuat.ac.jp (K.K.); 2 Department of International and Environmental Agriculture Science, Tokyo University of Agriculture and Technology, 3-5-8 Saiwaicho, Fuchu, Tokyo 183-8509, Japan; canticle2@gmail.com (C.P.O.); yosei@cc.tuat.ac.jp (Y.O.); 3 Department of Agricultural Economics and Agribusiness, University of Ghana, P.O. Box LG 64 Legon, Accra, Ghana; sylviasplash@yahoo.com; 4 Department of Crop Science, University of Ghana, P.O. Box LG 44 Legon, Accra, Ghana; camoatey@ug.edu.gh; 5 Institute of Agriculture, Tokyo University of Agriculture and Technology, 3-5-8 Saiwaicho, Fuchu, Tokyo 183-8509, Japan; agyeman@cc.tuat.ac.jp

**Keywords:** ethnobotanical survey, medicinal plants, Ejisu-Juaben municipality, indigenous knowledge, informant consensus factor, fidelity level

## Abstract

**Background:** The in-depth traditional knowledge of medicinal plants is at risk of extinction due to the dependency on oral transmission, and as such, there is an urgent need to document such knowledge. This study aimed to document indigenous uses of medicinal plants among community members in the Ejisu-Juaben Municipality. **Methods:** Data was collected in 2016 from community members and local herbalists in the Ejisu-Juaben Municipality through a semi-structured questionnaire. Statistical tools and ethnobotanical indices, i.e., informant consensus factor (ICF), fidelity level (FL), and use value (UV) were used to analyse the data. **Results:** One hundred and six medicinal plants belonging to 45 families were reported to cure 68 different human diseases. The most frequently used plant part in this study was the leaves (52%). Decoction (57.5%) and oral administration (58.3%) were the most utilised herbal preparation and administration route respectively. *Cleistopholis patens* had the highest UV (0.54) with pain & fevers and skin diseases having the highest ICF values (0.88 and 0.85 respectively). Furthermore, new medicinal uses of *Hilleria latifolia* and ten other species were recorded for the treatment of the traditional local disease, *aseram*. **Conclusions**: The current knowledge and uses of medicinal plants are still high in the study area based on the high degree of consensus among informants. This study could allow for the preservation of knowledge and biodiversity of medicinal plants, both of which are threatened with extinction.

## 1. Introduction

Human beings have depended on plants and their products directly for food, shelter, and clothing, and indirectly for their contribution to sustaining the ecosystem [1,2]. The efficiency of plant utilisation using available technology and knowledge would, therefore, be relevant for the continued survival of humanity [1]. The usage of medicinal plants was the primary approach to treating various ailments before the inception of Western medicine [1,3]. These medicinal plants are relatively freely available, resulting in an increasing demand for their utilisation to provide primary health care for many people [4,5,6]. In many developing African countries, about 80% of rural dwellers depend on traditional medicines for their primary health care [4]. There is, however, an increase in current research in the identification of active ingredients in medicinal plants, their role in the treatment of diseases, drug development, and preparations of herbal medicines [7,8,9,10]. Also, most of the medicinal plants that are very effective in the traditional medical system have some reported pharmaceutical effects [11,12,13]. Reliable information on useful traditional medicinal plants can be obtained through ethnobotanical studies. In many countries worldwide, including Ghana, people treat some diseases using traditional healthcare techniques [14,15,16,17,18]. In Ghana, for instance, the ratio of one physician per 1000 people is low (0.1), and this makes the role of traditional herbal practitioners very relevant [19,20]. Before becoming a British colony, indigenous medicines were associated with all facets of healthcare in Ghana [14]. However, the evaluation of medicinal plants and their products to ensure their safe use continues to be a significant challenge in the traditional healthcare system [19,21]. The Centre for Plant Medicine Research (CPMR) was set up by the Government of Ghana to conduct and promote various scientific activities that would improve herbal medicines. The activities of CPMR, as well as studies confirming the therapeutic evidence of herbal remedies [10,22,23], have further increased the usage of herbal medicines in Ghana. Nonetheless, the tradition of verbally transferring acquired indigenous knowledge on medicinal species to the next generation endangers traditional health knowledge among rural communities [24,25]. Regrettably, many Ghanaian traditional healers die with their wealth of secret indigenous knowledge [1]. The use of indigenous herbal medicines in health care has a strong basis in cultural and religious foundations existing in diverse ways among ethnic groups [26,27,28]. The documentation of indigenous knowledge via ethnobotanical studies will not only safeguard disappearing knowledge but will also help in the preservation and sustainable utilisation of medicinal plants [29,30,31]. Abbiw [1] reported on useful plants in Ghana including medicinal plants, while ethnobotanical studies on medicinal plants of Ghana have also been conducted in some parts of the country [8,12,15,23,32,33,34,35].

The population of Ejisu-Juaben Municipality comprises people who usually rely on forest and its products for most of their basic needs, including firewood, building materials, and medicinal plants. The indigenous medicinal knowledge shared among the large population of the local people, however, remains undocumented and risks disappearing. Although Asase and Asafo-Agyei [36] conducted a study on medicinal species used to treat malaria in some areas around the Bobiri Forest Reserve in the Ejisu-Juaben Municipality, no study has covered the entire municipality. However, there is limited information on the traditional use of plants to treat various diseases in the Municipality. Many potential medicinal plant uses in the entirety of the Ejisu-Juaben Municipality remain unexplored and are at high risk of eroding with time if accurate studies are not conducted. The present study seeks to identify and document medicinal species used to treat various diseases. This study, therefore, covers medicinal plant availability and explores traditional indigenous uses of plants in the treatment of various diseases.

## 2. Materials and Methods

### 2.1. Study Area

**Figure 1:**
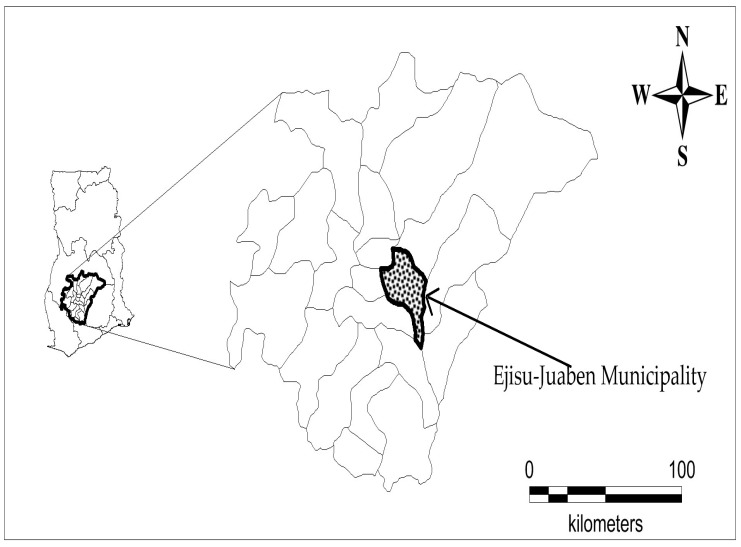
Map of Ghana showing Ejisu-Juaben Municipality.

The study was carried out in twenty communities in the Ejisu-Juaben Municipality, namely: Odoyefe, Juaben, Yaw Nkrumah, New Bomfa, Asotwe, Akyawkrom, Bonwire, Abetinim, Apemso, Nkyerepoaso, Kurofrom, Bowohomoden, Kwaso, Appiedu, Donaso, Achinakrom, Abenase, Kokode, Dumakwai, and Ofoase. The municipality stretches over a total land area of 582.5 km^2^, comprising about 10% of the entire Ashanti Region of Ghana. The Municipality lies within Latitudes 1°15′ N and 1°45′ N and Longitude 6°15′ W and 7°00′ W (Figure 1). The municipality has a bimodal rainfall pattern with semi-equatorial climate. In March-July and September-November, there are major (1200–1500 mm/year) and minor (900–1120 mm/year) rainfall periods respectively. Temperatures are lowest in August (around 25°C) and highest in March (around 32°C). The municipality is 240–300 m above sea level with generally undulating topography. The estimated population is 143,762 with agriculture (62.5%) as the predominant economic activity, followed by commerce and services (31.7%), and industry (6.8%). Among the farmers, crop farmers are the majority (94.1%) with the remaining 5.9% engaging in mixed farming [37]. Electricity accounts for about 69.4% of the main source of lighting, while wood (44.5%) is the major of fuel for cooking for many households. Boreholes, pipe borne water, and rivers are the main sources of water for the inhabitants. The Ejisu-Juaben Municipality is ethnically homogeneous, with Akans’ (82%) being predominant, and Christianity (84.1%) being the major religion [37]. In both 2012 and 2013, malaria, rheumatism, skin diseases, ulcers, respiratory tract infections, diarrhea, and anemia were among the top ten outpatient department cases in the municipality [37]. Even though there have been some advances in current health care in the municipality, some of the dwellers still rely on medicinal plants and traditional healers for their health needs. Consequently, the role of these traditional healers continues to remain relevant to local communities [38].

### 2.2. Questionnaire and Interviews with Local People

The survey was conducted from January–September 2016 to collect and document indigenous knowledge on the use of medicinal species in the Ejisu-Juaben Municipality. Semi-structured questionnaires (Supplementary File S1), field tours, personal interviews with 140 respondents based on standard ethnobotanical methods [39,40,41] were employed in this survey. The participants included various traditional healers and some local people who use medicinal plants in their households. We requested the voluntarily verbal consent of all informants in order to fulfil the seventh article of the Nagoya Protocol [42]. We assured the informants that the data obtained from this study would be used strictly for academic purposes. Opinion leaders and the local people were met, and the purpose of the survey was explained to optimize their participation. The questionnaire was designed in English and administered in *Twi*, the major language spoken in the study area. Some of the information recorded during this study included the bio-data of the informants, local name(s) of utilised plants, plant parts used, sources of plants, preparation and administration routes, diseases treated, etc. (Supplementary File S2). In total, 140 informants were interviewed, with each meeting usually lasting between 1–4 h, which included plant collection during field-walks. Two or three members from each household usually took part in the interview. The interview sessions usually involved 2–3 members from each household including the informant. Gifts were given to informants in the form of toilet soap and washing powder as part of local custom to show appreciation and to encourage the participation of others.

### 2.3. Botanical Identification

Informants aided the collection of voucher specimens following plant taxonomic method [43] and deposited them at the herbarium of the Centre for Plant Medicine Research, Mampong-Akropong. The identification of voucher specimens was aided by local botanists and also by comparing with already authenticated specimens at the herbarium. In this study, the nomenclatures of the species were verified using the database available online at the International Plant Names Index at www.ipni.org (accessed on 12 October 2018).

### 2.4. Data Analysis

#### 2.4.1. Statistical Analysis

Ethnobotanical data were then summarised and analyzed using Microsoft Office Excel^®^ (2010, Microsoft, Redmond, WA, USA) and IBM SPSS (version 20 software, IBM Corporation, Armonk, NY, USA) using descriptive statistical methods.

#### 2.4.2. Preference Ranking

Fifteen key informants carried out preference ranking [39] of the ten most available and preferred medicinal plant and ailments commonly treated. The shortlist was made based on plant availability and effectiveness in treating a particular disease. An overall rank value for a species was obtained by summing up the values assigned to each species across by all key informants. The species with the highest total rank value was ranked first, followed by the next species in that order. The level of destructive impacts was used for priority ranking [39] by the 15 key informants to rank the different factor considered as threats to the availability of medicinal species in the municipality. The informants assigned values 0–5 to each of the factors with 0 for no impact and 5 for the most destructive.

#### 2.4.3. Use Value (UV)

The relative importance attached to a given medicinal species in the study area was established using a quantitative method termed “use value” [44]. The use value (UV) was calculated using the Equation (1) below:

(1) $$\mathrm{UV}=\frac{\mathrm{Ui}}{N}$$

where UV denotes the use value of a particular medicinal species, Ui is the total number of use reports for a given medicinal plant by each informant, and N is the total number of respondents interviewed. A high use value for a species indicates the high use of the plant species, and a low use value indicates little use of the reported plant.

#### 2.4.4. Informant Consensus Factor (ICF*j*)

The data was checked for homogeneity in the use of medicinal plants in the treatment of ailment categories among the informants in our study area by calculating the informant consensus factor (ICF*j*) as used by Heinrich et al. [45]. An ailment category with low (near 0) ICF*j* value indicates that plants used in that category were randomly chosen, or that there was less exchange of information about their usage among informants. On the other hand, a high (near 1) ICF*j* value for a category means that the plants used were carefully selected in the community, or that there was shared knowledge on plant uses among informants [46]. The informant consensus factor (ICF*j*) was calculated using the Equation (2) below:

(2) $$\mathrm{ICF}j=\frac{\mathrm{Nur}j-\mathrm{Nt}j}{\mathrm{Nur}j-1}$$

where Nur*j* indicates the number of use reports in the ailment category *j*, and Nt*j* is the total number of taxa reported in the ailment category *j* by all respondents. A plant used to treat more than one disease in the same category was considered only once.

#### 2.4.5. Fidelity Level (FL)

The Fidelity Level [47] was calculated for each of the preferred medicinal plants to evaluate the importance of a species for a given purpose. This evaluation was based on key informants who cited specific plants in the treatment of particular ailments. The fidelity level shows the proportion of the respondents claiming to use a specific plant for the treatment of a specific ailment category. The following equation (3) was used:

(3) $$\mathrm{FL}=\frac{\mathrm{Ip}}{\mathrm{Iu}}\times 100$$

where Ip is the number of respondents who mentioned the usage of a plant to treat a particular disease, and Iu is the total number of respondents who cited the species for any use.

### 2.5. Ethics Approval and Consent

To participate in this survey, all participants gave their voluntary oral prior informed consent. No further ethics approval was required.

## 3. Results and Discussion

### 3.1. Demographic Features of Informants

**Figure 2:**
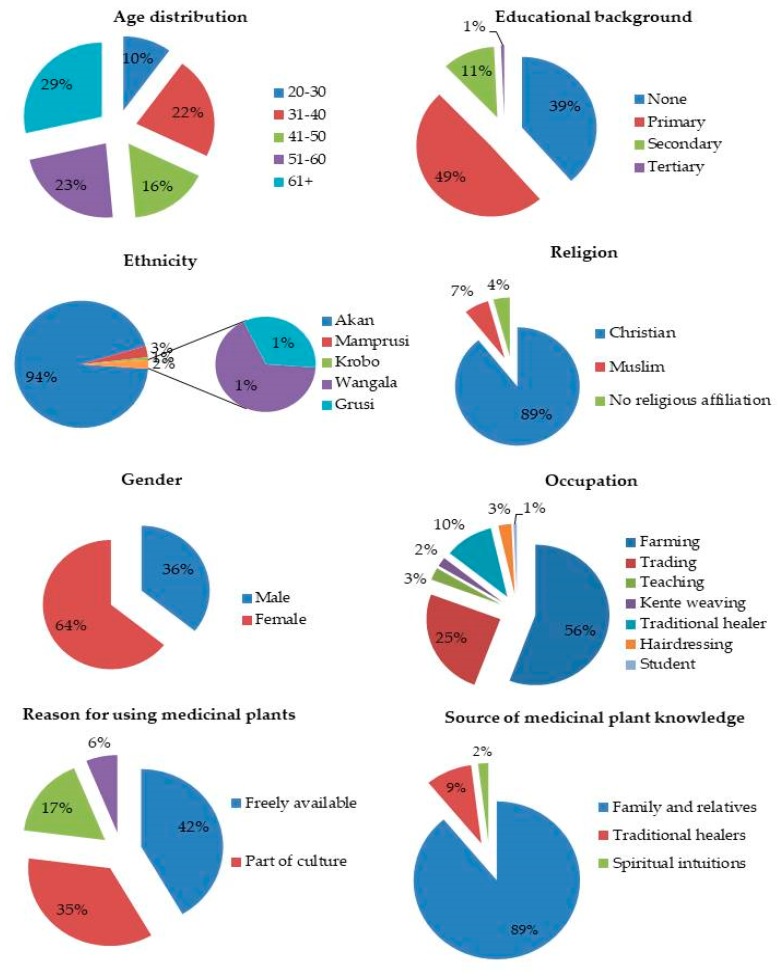
The demographic profile of the informants in Ejisu-Juaben Municipality.

During the survey, 140 respondents (males: 35.7% and females: 64.3%) aged between 28 and 85 were interviewed (Figure 2). The average ages of the informants were 63 and 68 for males and females respectively. The most acquired form of education was primary or basic education (49.3%), while 38.6% of the informants had no formal education. About 10.8% of the informants had secondary education, with only 0.7% having acquired tertiary education. Christians (88.2%) were the majority among the informants while Muslims were 6.4%. Akans were the predominant ethnic group (94.3%), with others like Mamprusi (2.9%), Wangala (1.4%), Grusi (0.7%), and Krobo (0.7%) represented in small fractions. The majority of the informants (89%) attained their indigenous medicinal plant knowledge orally through the experience of mostly close relatives. The practice of orally acquiring knowledge about medicinal plants was also reported by [48]. In Sisala, Northwest Ghana, Wodah and Asase [34], reported a similar mode of medicinal plant knowledge acquisition among traditional healers. Few of the respondents had received their indigenous knowledge directly from native healers (9%) and spiritual intuitions (2%). None of the informants obtained their knowledge through formal training. Results from our study showed that most of the respondents (41.6%) used medicinal plants because of their free availability, while 35.4% of the participants claimed that their use of medicinal plants was part of their culture. However, 16.8% claimed that western medicine could not cure some diseases, while only 6.2% indicated western medicines resulted in too many side effects. Contrary to these findings, the majority of medicinal plant users in Rodrigues Island, Mauritius, claimed that they used medicinal plants as part of their culture [49]. There is, however, a report that medicines derived from plants are relatively safer than their synthetic counterparts, thus giving abstruse therapeutic benefits and less expensive treatment [50].

### 3.2. Medicinal Plant Diversity and Distribution

In the present study, we documented 106 medicinal plant species belonging 45 plant families, used in the treatment of 68 different human diseases in the Ejisu-Juaben Municipality. Medicinal plants mentioned by at least three respondents during the survey were considered. Almost all the reported species in the study had the same local name throughout the understudied communities in the Ejisu-Juaben Municipality. This gives a strong indication of homogeneity of vernacular nomenclature among inhabitants in the municipality, which is an essential aspect of indigenous knowledge transfer. The Fabaceae plant family (12) had the highest number of species, followed by the Euphorbiaceae (9), Asteraceae (6), Poaceae (6), and Malvaceae (5) families.

The families of Apocynaceae, Bignoniaceae, Meliaceae, and Rutaceae (with four species each) together with Amaranthaceae, Anacardiaceae, Combretaceae, Rubiaceae, and Solanaceae (with three species each) were also represented (Table 1). The other families had two or only one species recorded in this study. In other parts of Ghana, Fabaceae had high species representations among medicinal plants [15,34,35].

**Figure 3:**
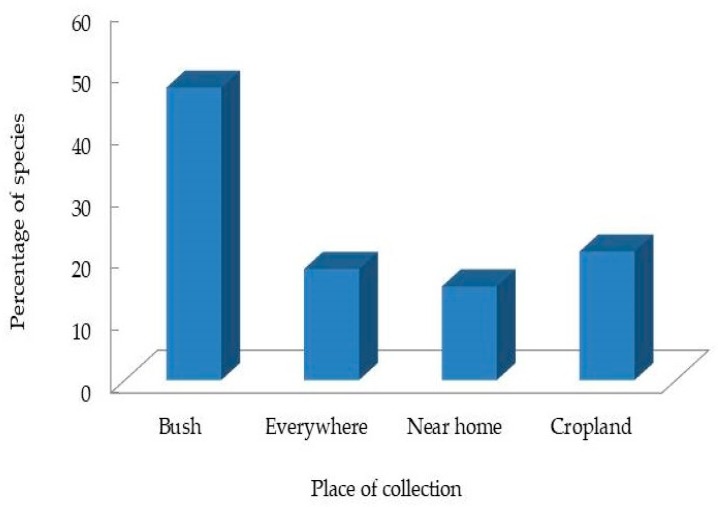
Places of collection of medicinal plants cited in this study.

In Western Ghat, India [51], Djibouti [52], Burkina Faso [53], and Uganda [17], Fabaceae was also the predominant plant family among the documented medicinal plants. For each of the medicinal plants recorded in this study, scientific name, voucher specimen number and local name, plant parts used, traditional medicinal use(s), mode of preparation and administration routes, and use values were given. About 47% of the reported medicinal species used in the study area were collected from the bush (Figure 3). The informants also collected some of the reported species (21%) from croplands, and a few of the common species (18%) could be collected from virtually everywhere on the landscape. The majority of the species collected from croplands were cultivated plants grown extensively as food but also used for their medicinal properties when required. Plants used among traditional healers in Sisala were also mostly collected in the wild (55%) and from similar Ecozones found in this study [34]. In contrast, the majority of medicinal plant users in places surrounding the Bobiri Forest Reserve in the Municipality collected medicinal plants from their immediate surroundings [36].

### 3.3. Ailments Treated and Preferred Medicinal Plants Species

Through the native knowledge of medicinal plant users in the Ejisu-Juaben Municipality, information about the treatment of 68 different diseases (Table 2) were obtained. A wide range of ailments were treated by the species documented in this study, varying from one to seven per plant. The range of diseases treated by plants mentioned in this study expanded from one to seven diseases per plant. *Momordica charantia* (seven health conditions), followed by *Cleistopholis patens*, *Newbouldia laevis*, *Ocimum gratissimum*, and *Alchornea cordifolia* (six health conditions each) treated the highest number of diseases. In Algeria [54], Uganda [17], and Ethiopia [55], single plants were reported for the treatment of multiple diseases among informants. Some of the local healers indicated two traditional infant diseases (*aseram* and *asabera*, as they are known in the *Twi* dialect) that have no clear biomedical equivalent. They claimed that these diseases are transmitted “spiritually” and considered as *not-for-hospital* diseases. A similar infant health condition, “*Evil eye*”, described as an ailment of babies, or adults and in some occasions unborn babies as well, could be contracted when a person admired them ardently while secretly being jealous [56].

According to the informants, “*aseram*” is characterized by green/black visible veins, a big head, and lean growth of the baby, whiles “*asabera*” has an extremely hot body, pale eyes, and frequent green, foamy bloody stools as major symptoms. Other childhood illnesses (*puni, enfire yare*, and *ananosono*) have also been identified and categorized as *not-for-hospital* diseases in Ghana [57,58]. Obiri and Addai [59] reported the use of *Crotalaria* spp for the treatment of such traditional infant ailments. In this study, informants mentioned 13 medicinal species for the treatment of such childhood diseases, which, they perceive, cannot be treated at health facilities. Among these species, *Hilleria latifolia* (FL = 100%), *Tapinanthus bangwenis* (FL = 82.4%), *Mallotus oppositifolius* (FL = 75%), and *Lannea welwitschii* (FL = 66.7%) were commonly used to treat “*aseram*” while *Eclipta alba* (FL = 58.8) and *Vernonia amygdalina* (FL = 13%) were used in treating “*asabera*”. *Azadirachta indica* was the most highly-ranked species in the study area by key informants (15 traditional healers). *A. indica* was mentioned mainly for the treatment of malaria.

The ranking of the ten preferred medicinal species according to our key informants, based on plant availability and effectiveness in treating the mentioned diseases, is in Table 3. Four out of the ten preferred medicinal species were used in the treatment of malaria, and this indicated the importance of the disease and such plants in the municipality. *Vernonia amygdalina* ranked 4th in this study and is used to treat malaria, typhoid, “*asabera*” (a non-communicable spiritual ailment affecting children and causing severe fever); it was the most highly ranked species in Uganda for the treatment of malaria [17].

### 3.4. Plant Parts Used and Growth Forms of Medicinal Plants 

**Figure 4:**
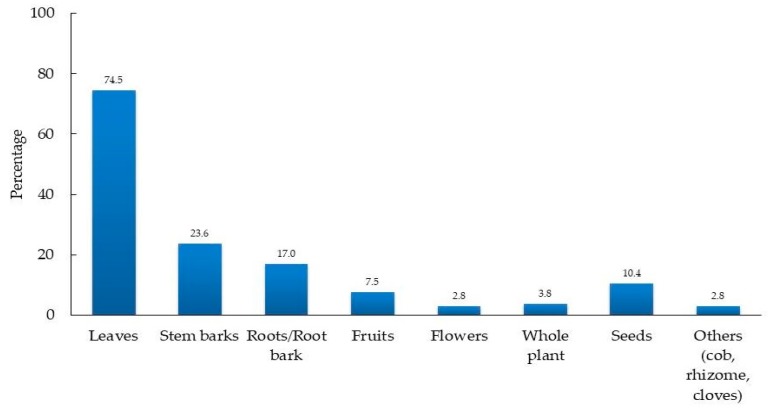
Plant parts used by informants for medicinal purposes.

Medicinal plants users including traditional healers in the study area used different medicinal plant parts for their herbal remedies preparations (Figure 4). Most of the herbal medicines were prepared using leaves (52%) and bark of stems (17%). Root/root barks (12%) were also commonly used in herbal medicine preparation. The seeds, fruits, whole plant, flowers, and others plant parts like rhizome, cob, and cloves were cited less frequently. Inhabitants in other parts of Ghana including the Dangme West District [8], Sisala East and West districts [34], surrounding areas of Bobiri Forest Reserve [36], and Kpando Municipality [60] also preferred the use of leaves in herbal medicine preparation. Also, a similar practice is reported in neighboring countries including Cote d’Ivoire [61], Togo [62], and Burkina Faso [53]. This high preference for leaves during plant medicine preparations was due to the availability and ease of collection [63,64].

**Figure 5:**
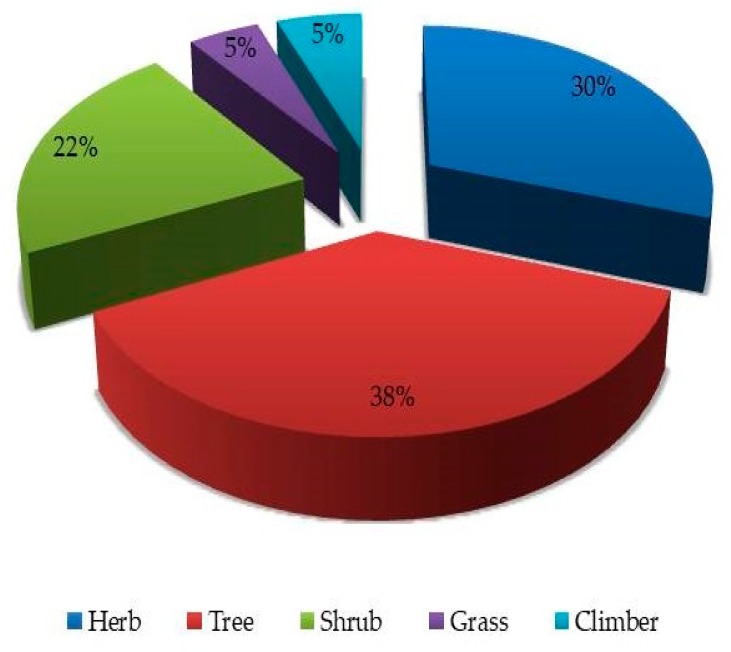
The growth form of medicinal plants documented in this study.

Additionally, photosynthetic activities mostly occur in the leaves leading to the production of most bioactive substances that may result in the curative effects of medicinal species [65]. In contrast, roots were reported as the most exploited plant part in the Oshikoto Region in Namibia [66] and Eastern Nepal [67]. This discrepancy in plant parts usage may be the result of culture, species diversity, and bioactive substances found in the plant parts in those areas. However, the plant part(s) collected for herbal medicine preparations, as well as the frequency of harvesting and the amount harvested, could have an impact on the harvested plant [68]. The use of roots and the barks of roots and stems in the municipality make those species vulnerable to overexploitation. Some respondents cited the use of multiple plant parts for the preparation of herbal remedies. Various parts of *R. vomitoria, C. patens* and *M. paradisiaca* were combined during the preparation of herbal medicines. Nonetheless, such medicinal plants could be liable to overexploitation if such practice continues. The use of leaves, however, could be less destructive to plants when the frequency of harvesting and amounts collected are regulated. The results of this study show that the inhabitants of the study area mostly utilized trees (38%) for the treatment of various ailments and disorders, followed by herbs (30%), shrubs (22%), climbers (5%), and grass species (5%) as shown in Figure 5. In contrast, most of the medicinal plants in Cameroon [69], Pakistan [70], and Uganda [17] were herbs. Shrubs were, however, the predominant growth form of medicinal species in Ethiopia [71] and Djibouti [52]. The majority of medicinal plants cited in this study were either weeds (34%) or species in the wild (29%). This indicated that the majority of the medicinal plants in the understudied municipality have less protection from over-exploitation, and this does not encourage their conservation.

### 3.5. Herbal Medicine Preparation and Route of Administration of Herbal Remedies

**Figure 6:**
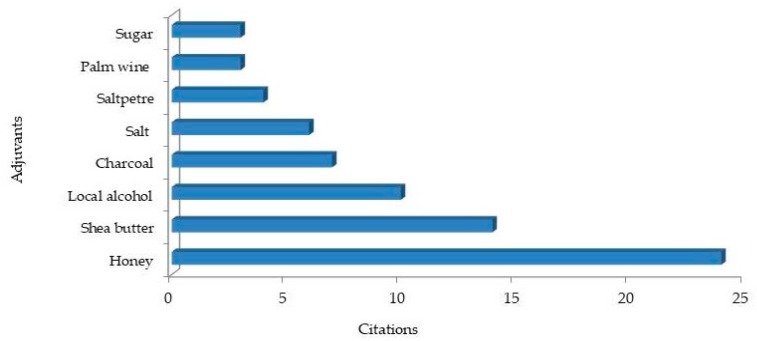
Non-plant ingredients added to medicinal plants used by local healers and other medicinal plant users.

The respondents employed various herbal preparation methods to treat ailments in the Ejisu-Juaben Municipality. Our results showed that most of the herbal remedy preparations (57.6%) were formulated from a mixture of other plants or non-plant ingredients. A similar observation was made among traditional healers in Mascara (Algeria), where 51.4% of the reported species were used as mixtures with other materials [72]. It is believed that the use of medicinal plant mixtures can provide a positive synergic effect and also improve the taste or smell of the mixture [73,74]. Among the 89 species that were cited in mixed herbal formulations, 25.8% were mixed with adjuvants such as honey, salt, Shea butter, alcohol, saltpetre, or charcoal (Figure 6). Honey, Shea butter, and the local alcohol “*akpeteshie”* were the most cited adjuvants. The use of honey, milk, sugar, and other non-plant adjuvants in the preparation of herbal medicines have been reported in other studies [54,72,75]. Salt was usually used in our study area for herbal preparations against toothache and sore throat. A similar observation has been reported among traditional healers in the surroundings of Mabira Central Forest Reserve in Uganda [17]. Some of the respondents (traditional healers especially) revealed that the effectiveness of their herbal preparations would diminish if they showed the exact adjuvants added to their herbal formulations. It is a common practice for some traditional herbalist to guide their native knowledge by creating mystery around herbal formulations and dosages administered to patients [14,76]. Secrecy is considered one of the primary obstacles facing the spread of traditional indigenous knowledge among inhabitants. This mystery of secrecy surrounding medicinal plants also blocks any attempts to reconcile the divide between herbalists and conventional medicinal system [77].

**Figure 7:**
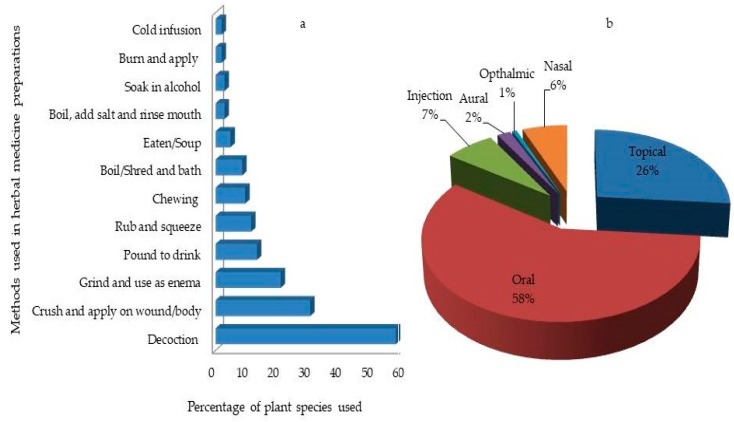
Mode of preparation (**a**) and administration (**b**) of medicinal plants collected in the Ejisu-Juaben Municipality.

Herbal remedy preparation methods such as decoction (58%) crushing (30%), grinding to use as an enema (21%), pounding to drink (13%), rubbing and squeezing (11%) were used (Figure 7a). Chewing, bathing, eating, soaking in alcohol and mouthwashes were cited less. Oral administration (58%) was the frequently cited mode of administration (Figure 7b). This was followed by topical applications such as body massage, smearing as body lotion, or tying onto the wound, bath (26%). In other parts of Ghana, oral intake was reported as the major route of herbal remedies administration [8,15,23,34,36] as it was in other countries [17,26,28,55]. Furthermore, the use of enema (29%) was also a common recommendation among the respondents in the municipality. In Ghana, the use of enemas in traditional healthcare delivery is a common cultural practice. This was mostly prescribed for complications related to pregnancy, early child care, constipation, and other stomach related complications [78].

### 3.6. The Use Value (UV)

The use value (UV) indicates the relative importance of medicinal species to the plant users (Table 2). In this study, the UV ranged from 0.02 to 0.54, with the species having the highest UV being *Cleistopholis patens* (0.54). Other species such as *Ocimum gratissimum* (0.37), *Alstonia boonei* (0.36), *Senna occidentalis* (0.35), and *Azadirachta indica* (0.33) also had high use value. These values are high compared to the use value indices reported for some medicinal species from Burkina Faso [79]. The high use value of each of these plants indicates that they are the best known, recommended, and utilized by the respondents, and this denotes the importance placed on these species. A high UV of plants can also indicate their abundance in the specific study area [51]. The lowest UVs (0.02) were reported among *Lannea welwitschii*, *Elaeis guineensis*, *Baphia nitida*, *Mimosa pudica*, *Griffonia simplicifolia*, and 15 other species. The reason for this low UV can be due to the limited information on the medicinal uses of these plants possessed by the informants.

### 3.7. The Informant Consensus Factor (ICF)

The 68 different health conditions were grouped into 15 ailment categories in this study: pains & fever, skin diseases, erectile dysfunction & impotence, and gastrointestinal system diseases. And also, gynecological issues and child care, respiratory tract infections, STDs & venereal diseases, muscular skeletal problems, poisonous animal bites, blood problems/tonic/stimulant, arthritis & inflammation, eye and ear infections, neurological & nervous system disorders, cardiovascular diseases, and other ailments (bedwetting, rituals, and to stop alcoholism). Informant consensus factor (ICF) was calculated to check the uniformity of the data provided by the respondents on the medicinal plants documented and the ailment categories treated (Table 4). Pains & fever ailments category had ICF value of 0.88 and was the highest recorded in this study. The highest number of medicinal plants was cited for the treatment of pains and fever disease category (migraines, headaches, fever, malaria, abdominal pain, waist and body pains) for which 48% of total species (51 species) were mentioned. Out of the 51 species mentioned in this category, most (53%) were involved in the treatment of malaria. Malaria remains the leading disease among the top 10 outpatient department (OPD) attendance in the Ejisu-Juaben Municipality [38]. Species like *Cleistopholis patens* (68 citations), *Azadirachta indica* (46 citations), and *Vernonia amygdalina* (15 citations) frequently mentioned during the survey have also been cited in other parts of Ghana for the treatment of malaria [8,15,33,36].

Similar to the findings in this study, the ICF value of malaria, fever, and headache ailment category (0.82) was the highest recorded among informants in Wonago Woreda, Ethiopia [80]. Plants used in Maonan communities in China [81] to treat fever and malaria had a high reputation and also had a high ICF value (0.73). The next highest ICF values were recorded for skin diseases, erectile dysfunction & impotence, gastrointestinal system diseases, and gynaecological issues & child care with values of 0.85, 0.81, 0.80, and 0.74 respectively. Skin diseases had high ICF values in other studies conducted in India [51], Rodrigues Island, Mauritius [49] and Algeria [54], with values of 0.69, 0.98 and 0.75, respectively. Although skin diseases had less number of taxa (29), it had higher ICF value than gastro-intestinal system diseases (50 taxa). The high ICF for skin diseases could be due to better communication and information distribution among respondents on plants used to treat skin diseases resulting from high disease prevalence. Similarly, there was a better understanding among informant on plants used for erectile dysfunction & impotence (ICF = 0.81). Although a relatively high ICF value (0.68) has been reported for similar ailment category in China [81], very low ICF values were reported for this category in Uganda [17] and in Iraq [28] and with ICF values of 0.2 and 0 respectively. The usage of a substantially higher number of plants species (50 species) to treat diseases in the gastrointestinal ailment category could mean the high occurrence in the area, likely due to low level of sanitation. Other studies in Djibouti [52], Ethiopia [55], Algeria [54], and Nepal [67] also reported high ICF values for gastrointestinal ailment category with ICF values of 0.8, 0.75, 0.87, and 0.78 respectively.

### 3.8. The Fidelity Level (FL) 

Fidelity level (FL) was calculated for the medicinal plants cited by the informants based on their relative effectiveness to treat key ailment mentioned (Table 2). Both *A. indica* and *T. grandis* had fidelity level of 100%. Other studies in some parts of Ghana reported high ranks for *A. indica* and *T. grandis* for treating malaria [8,33,36]. In countries like Uganda [82], Nigeria [83], and Kenya [84], *A. indica* was cited for malaria treatment. Both the tablet and suspension from leaves and bark of *A. indica* have been reported with antimalarial activity, with the leaf suspension being more effective than the bark [85]. Aside from malaria, *A. indica* is also used to treat fever, typhoid, rheumatism, skin rashes, and urogenital related worms in other parts of Ghana [23,86]. *T. grandis* is used in some parts of Ghana [36,87], Nigeria [88], and Cote d’Ivoire [89] for treating malaria. The anthraquinones derivatives from the leaves of *T. grandis* have shown significant in vitro antimalarial activity [90]. In Togo, *T. grandis* is being used to treat malaria and diabetes mellitus [91,92], hair loss and dandruff in India [51], and anemia in Benin [93]. Medicinal plants with high FL values could be prospective candidates for advanced pharmacological research to validate in vitro action [52]. Other species with high FL values in the study area were also utilized in some parts of Ghana or other countries to treat the same or different health conditions. *Cleistopholis patens* (FL = 91%) had been utilized in the treatment of malaria in some parts of Ghana [36,94] and Cameroon [95]. *Senna occidentalis* (FL = 80%) is used to treat malaria in other parts of Ghana [94]. It has also been used to manage constipation, indigestion, and hypertension in China [81], infertility in Nigeria [96], and stomach complaints in Brazil [97]. *Vernonia amygdalina* (FL = 65%) was vastly desired in Uganda to treat malaria and convulsions [17] and for treating impotence and dandruff Ethiopia [55]. In Rodrigues Island, Mauritius, the fruit of *M. charantia* is consumed as a salad and was cited to be active in the treatment of diabetes mellitus and hypercholesterolemia [49]. Moreover, the leaves of *M. charantia* are used in the production of juice in Bangladesh for the treatment of helminthiasis and diabetes [98]. In Uganda [17], *A. boonei* was used in treating malaria, hemorrhoids and malaria in Nigeria [83,99,100]. *Senna alata* was also used to treat skin diseases in Nigeria [101] and Bangladesh [102]. *Phyllanthus urinaria* was cited to be effective against diarrhea in Trinidad and Tobago [103], malaria in Thailand [104], and dental ulcer in China [105]. The low FL values of *P. urinaria* (38%) and *Alchornea cordifolia* (48%) for the treatment of piles and ulcer are likely due to the low occurrence of these ailments and hence the narrow distribution of information about their management in the study area.

### 3.9. Threats to Medicinal Plants and Conservation Practices

The study established that the constant availability of medicinal species in the study area is faced with some threats in their natural habitats. The key informants who collect medicinal plants on a regular basis claimed that plants were previously abundant in the natural vegetation around farmland, home compounds, roadsides, and riversides. However, none of the key informants had a garden specifically for any of the medicinal plants they cited. The Bobiri Forest Reserve is the only conservation effort that protects plants (including some medicinal plants) and animal species in the municipality [38,106,107]. Expansion of agricultural activities was ranked as the biggest risk to the continued availability of medicinal floras in the study area. Although most of the inhabitants of the municipality are farmers, the introduction of the President’s Special Initiative (PSI) on oil palm, coupled with the siting of the Juaben Oil Mills in the municipality had increased the area of land under oil palm plantation [108]. Land area under tree crops is also increasing leading to the clearing of natural vegetation in the Municipality [109]. The threat of agricultural growth was followed by drought, excessive harvesting, and bushfires in that order (Table 5). Agricultural expansion has also been reported to be the biggest threat to the survival of medicinal plants in studies conducted in Ethiopia [55,80,110] and Uganda [80]. Contrary to these results, bushfires and drought were the major threats identified by other informants in different parts of Ghana [34,36], with deforestation being the most mentioned threat in Ethiopia [71]. Overgrazing and floods were ranked as low threats, and this may be due to the low interest in large-scale livestock rearing and the undulating topography of this study area respectively. The Forestry Research Institute of Ghana (FORIG) has made efforts, to develop propagation methods for some of the endangered species in the municipality. However, propagation methods have been documented for only ten medicinal plant species [111].

## 4. Conclusions

The present study indicates that medicinal plants are still relevant in primary healthcare delivery among the inhabitants of Ejisu-Juaben Municipality. The Fabaceae family was found to have had the highest number of species used in treating these ailments. The study documented 106 medicinal species used to treat 68 diseases in the study area. Also, new traditional herbal use of *Hilleria latifolia, Tapinanthus bangwenis, Alternanthera pungens, Rauwolfia vomitoria, Bidens pilosa, Newbouldia laevis, Carica papaya, Kalanchoe integra, Momordica charantia, Mallotus oppositifolius, Lannea welwitschii, Eclipta alba* and *Vernonia amygdalina* to treat “*asabera”* and “*aseram*” are reported for the first time in this study.

*Asabera* and *aseram* are local disease deemed *not-for-hospital* and local herbalists treat them by using herbal medicine in the study area. Although agricultural expansion could lead to food security, local healers have warned that it is a significant threat to the continued availability of medicinal plants in the Ejisu-Juaben Municipality. The key informants also listed drought and excessive harvesting as threats to medicinal plants. Also, most of the medicinal plants used in our study area were collected from the wild bushes, and are not under cultivation. This documentation could contribute to safeguarding indigenous knowledge on medicinal plants, which is still verbally transferred among local people. The therapeutic claims of useful medicinal species should be further investigated to identify the bioactive compound(s) that could further be used to develop new drugs.

## Figures and Tables

**Table 1:** Taxonomic diversity of medicinal plants in the Ejisu-Juaben Municipality.

Plant Families	No. of Plant Genera	Percentage of Genera	No. of Plant Species	% of Species
Fabaceae	9	9.38	12	11.3
Euphorbiaceae	8	8.33	9	8.49
Asteraceae	6	6.25	6	5.66
Poaceae	6	6.25	6	5.66
Malvaceae	5	5.21	5	4.72
Apocynaceae	4	4.17	4	3.77
Bignoniaceae	4	4.17	4	3.77
Meliaceae	4	4.17	4	3.77
Rutaceae	2	2.08	4	3.77
Amaranthaceae	3	3.13	3	2.83
Anacardiaceae	3	3.13	3	2.83
Combretaceae	2	2.08	3	2.83
Rubiaceae	3	3.13	3	2.83
Solanaceae	1	1.04	3	2.83
Other 31 families	36	37.5	37	34.9
Total	96	100	106	100%

**Table 2:** Medicinal plants, their habit, parts used, ailments treated, cultivation status, method of preparation and administration in the Ejisu-Juaben Municipality.

Plant Family, Scientific Names, Voucher No.	Local Names	Parts(s) Used	Ailments(s) Treated	Mode of Preparation (Administration)	Ethnobotanical Indices		
					FL (%)	UV	RFC
**Acanthaceae**							
*Justicia flava* Vahl. CPMR 4089	Afama	L	Malaria, **Diarrhoea**, Piles	Decoction (Oral)	60	0.14	0.13
			Swollen joint	Grind (Topical)			
**Amaranthaceae**							
*Alternanthera pungens* Kunth CPMR 4090	Nkasɛɛ nkasɛɛ	L	Constipation, *Aseram*, Headache	Grind (Enema)	81.8	0.08	0.06
*Amaranthus spinosus* L. CPMR 4092	Nantwibini	Wp	Malaria, Dewormer, Fever	Decoction (Oral)	47.1	0.12	0.06
			Delayed baby walking	Crush (Bath/enema)			
*Pupalia lappacea* (L.) A Juss. CPMR 4091	Apɔsɔmpɔ	L	Boils	Burn (Topical)	100	0.04	0.02
**Anacardiaceae**							
*Lannea welwitschii* (Hiern) Engl. 4093	Kumnini	SB, L	Child fever, *Aseram*	Boil (Bath)	66.7	0.02	0.01
*Anacardium occidentale* L. CPMR 4094	Ateaa	L	Pregnancy care	Decoction (Oral)	100	0.04	0.02
*Mangifera indica* L. CPMR 4095	Amango	SB	Diarrhoea	Paste (Enema)	50	0.09	0.1
		L	Cough, Bedwetting, Low sperm count	Decoction (Oral)			
**Annonaceae**							
*Cleistopholis patens* (Benth.) Engl. & Diels CPMR 4070	Ngone nkyene	L/SB, F	Stomach ache, Fever Malaria, typhoid	Decoction (Oral)	90.7	0.54	0.37
		R/SB, Se	Hernia, Piles	Grind (Enema)			
**Apocynaceae**							
*Alstonia boonei* De Wild. CPMR 4096	Nyamedua	SB	Measles, Chicken pox, Boils, Shingles	Crush (Topical)	70	0.36	0.22
			Malaria	Decoction (Oral)			
*Funtumia elastica* (Preuss) Stapf. CPMR 4097	Funtum	SB	Stroke,	Infusion (Oral)	66.7	0.02	0.01
			Stop alcoholism	Soak in alcohol			
*Rauvolfia vomitoria* Afzel. CPMR 4081	Kakapenpen	L, R	*Aseram*, Malaria, Body pains	Decoction (Oral)	41.7	0.09	0.09
		SB	Piles, Chicken pox	Grind (Topical)			
**Asclepiadaceae**							
*Secamone afzelii* (Roem. & Schult.) K.Schum. CPMR 4098	Kwantemaa	L	**Severe skin rashes**	Grind and apply (Topical)	100	0.03	0.01
*Parquetina nigrescens* (Afzel). Bullock	Abakamo	SB	Wounds, Snake bite	Grind (Topical)	40	0.04	0.02
CPMR 4102		L	Family planning	Paste (Topical)			
		SB	**Piles**	Squeeze (Oral)			
**Araceae**							
*Xanthosoma mafaffa* Schott CPMR 4099	Mankani	L	Snakebite, **wounds**	Squeeze (Topical)	60	0.07	0.04
			Blood Tonic	Cooked (Oral)			
**Arecaceae**							
*Cocos nucifera* L. CPMR 4100	Kube	L, R,	Ulcers, Piles	Decoction (Oral)	45.5	0.08	0.04
		R	**Erectile dysfunction,**	Infusion (Oral)			
		R	Toothache, Bad breathe	Decoction (Oral)			
*Elaeis guineensis* Jacq. CPMR 4101	Abɛ	R	**Stroke**	Grind (Topical)	100	0.02	0.02
**Asphodelaceae**							
*Aloe cf. tenuifolia* Lam. CPMR 4103		L	Appetizer, **Skin Rashes**	Decoction (Oral/Topical)	75	0.06	0.03
**Asteraceae**							
*Melanthera scandens* Schu, Nach. & Thonn. CPMR 4077	Mfofo	L,	**Wounds**	Paste (Topical)	100	0.04	0.01
*Vernonia amygdalina* Delile CPMR 4088	Awɔnwono	L	Diarrhoea, Typhoid, **Malaria**	Decoction (Oral)	65.2	0.16	0.24
			*Asabera*	Crush (Topical)			
*Taraxacum officinale* F.H.Wigg. CPMR 4104	Dandelion	L, R	Blood tonic, Fever, Ulcer	Eaten/Decoction (Oral)	42.9	0.05	0.04
*Ageratum conyzoides* L. CPMR 4105	Guakro	L, R	**Eye disease,**	Rub and squeeze (Topical)	42.9	0.05	0.01
			Constipation	Grind (Enema)			
*Eclipta alba* Hassk. CPMR 4106	Ntum	L	***Asabera*, Catarrh, Malaria**	Grind and squeeze (Nasal)	50	0.04	0.11
*Bidens pilosa* L. CPMR 4107	Gyinantwi	L	Typhoid, Malaria,	Decoction/Paste (Oral/Topical)	40	0.04	0.01
			**Itchy ear**, *Aseram*				
**Bignoniaceae**							
*Kigelia africana* (Lam.) Benth. CPMR 4074	Nufutin	SB	**Stomach ache**	Decoction (Oral)	100	0.04	0.02
*Spathodea campanulata* P. Beauv. CPMR 4084	Kuakuanisuo	L, SB	Typhoid, **Malaria**	Decoction (Oral)	66.7	0.15	0.11
			Wounds	Grind (Topical)			
*Crescentia cujete* L. CPMR 4108	Kɔntoa	L	Fever, **Pregnancy care**	Decoction (oral)	57.1	0.05	0.01
*Newbouldia laevis* (P.Beauv.) Seem.	Sɛsɛmasa	L	Stomach upset, Waist pain, *Aseram*	Paste (Enema)	26.7	0.11	0.09
CPMR 4109		SB	Pregnancy care, **Cough**, High blood pressure	Raw (Oral)			
**Boraginaceae**							
*Heliotropium indicum* L. CPMR 4110	Akonfɛm atikɔ	L	Headache	Crush (Topical)	33.3	0.09	0.08
			Stomachaches, **Pregnancy care**, Headache	Shred (Topical)			
			Anaemia	Decoction (Oral)			
**Bromeliaceae**							
*Ananas comosus* (L.) Merr. CPMR 4111	Abrɔbɛ	L	Worms, Itching ear	Rub (Oral/Enema)	62.5	0.06	0.04
		F	**Malaria**, Severe fever	Decoction (Oral)			
**Caricaceae**							
*Carica papaya* L. CPMR 4112	Brɔferɛ	L	**Malaria**	Cook (Oral)	30.6	0.26	0.25
		R, Se	Ease delivery, *Aseram*, Dewormer	Raw (Oral)			
**Combretaceae**							
*Combretum smeathmannii* G. Don. CPMR 4113	Hwirɛmoo	L	**Migraine**, Baby delivery	Raw/rub (Oral)	60	0.04	0.03
*Terminalia catappa* L. CPMR 4114	Abrɔfo nkateɛ	L	Severe fever, **malaria**	Decoction (Oral)	66.7	0.04	0.04
*Terminalia ivorensis* A.Chev. CPMR 4115	Ɛmirɛ	SB	Piles	Powder (Oral)	100	0.04	0.03
**Compositae**							
*Chromolaena odorata* (L.) R.M.King & H.Rob. DC. CPMR 4116	Akyeampong	L	Blood clotting, **Wounds**	Squeeze/rub (Topical)	61.5	0.09	0.07
**Crassulaceae**							
*Kalanchoe integra* Kuntze. CPMR 4118	Egorɔ	L	**Headache**, *Aseram*, Cold, Phlegm	Squeeze (Topical)	61.9	0.15	0.14
		Wp	Stroke	Decoction (oral)			
*Bryophyllum pinnatum* (Lam.) Oken CPMR 4117	Taa meawu	L	Abdominal pain	Decoction drunk	100	0.04	0.06
**Cucurbitaceae**							
*Momordica charantia* L. CPMR 4078	Nyanya	L	**Abdominal pains**, fever, measles, Gonorrhoea, headache	Grind (topical)	60.7	0.2	0.15
			Snakebite, *aseram*	Decoction (oral)			
				Squeeze (topical)			
**Euphorbiaceae**							
*Mareya micrantha* (Benth.) Müll.Arg. CPMR 4119	Odubrafoɔ	L	**Waist pains**	Paste (Enema)	100	0.04	0.03
*Ricinodendron heudelotii* (Baill.) Heckel CPMR 4120	Ɔnwama	L	**Malaria**,	Decoction (Oral)	60	0.04	0.04
		L, R	Waist pains	Grind (Enema)			
*Phyllanthus muellerianus* (Kuntze.) Exell. CPMR 4121	Awobɛ	L	**Wounds**	Grind (Topical)	100	0.03	0.01
*Mallotus oppositifolius* Müll.Arg.	Nyanyafurowa	L	*Aseram*, Waist pains,	Grind (Topical)	75	0.06	0.06
CPMR 4076			**Constipation**	Paste (Enema)			
*Manihot esculenta* Crantz CPMR 4122	Bankye	L, Se	Fever	Decoction (Oral)	44.4	0.06	0.06
			**Blood clotting**, Wounds	Grind (Topical)			
*Alchornea cordifolia* (Schumach. & Thonn.) Müll.Arg. CPMR 4065	Ogyama	L,	Constipation	Grind (Enema)	31	0.21	0.16
		SB	Fever, **Malaria**	Decoction (Oral)			
		SB	Wounds, **Piles**	Rub (Topical/enema			
*Phyllanthus urinaria* L. CPMR 4080	Bɔwomaguwakyi	L	Blood pressure, **malaria**	Decoction (Oral)	37.9	0.21	0.16
			Sore throat, Ulcer	Raw (Oral)			
*Euphorbia hirta* L. CPMR 4071	Kakaweadwe	L	Wounds	Grind (Topical)	100	0.02	0.01
*Jatropha curcas* L. CPMR 4123	Nkrangyedua	R,	Severe cough	Crush (Oral)	30.8	0.09	0.09
		L	**Wounds**, Piles	Paste (Enema)			
		L, R	Toothache	Poultice (Buccal)			
**Fabaceae**							
*Senna occidentalis* (L.) Link CPMR 4082	Nkwadaa brodeɛ	L	**Stomachaches**, Malaria	Decoction (Oral)	79.6	0.35	0.21
		R	Serious vomiting, Cough	Raw (Oral)			
*Tetrapleura tetraptera* (Schum. & Thonn.) Taub. CPMR 4087	Prɛkɛsɛ	F	**Typhoid**, Asthma, Blood tonic	Decoction (Oral)	50	0.04	0.06
*Baphia nitida* Lodd. CPMR 4068	Ɔdwono	L	Wounds	Crush (Topical)	100	0.02	0.02
*Acacia pennata* Willd. CPMR 4124	Nwerɛ	L	Skin rashes	Grind (Topical)	75	0.03	0.02
			**Fever**	Decoction (Oral)			
*Griffonia simplicifolia* (DC.) Baill.	Kagya	Se	**Stomachaches**	Raw (Oral)	100	0.02	0.02
CPMR 4073							
*Senna alata* (L.) Roxb. CPMR 4083	Sempe	L	**Eczema**, Ringworm, Dandruff, Skin rashes	Grind (Topical)	33.3	0.28	0.16
			Stomachaches, Fever				
				Decoction (Oral)			
*Mimosa pudica* L. CPMR 4125	Mumuanka	L	**Wounds**	Grind (Topical)	100	0.02	0.02
*Albizia ferruginea* (Guill. & Perr.) Benth. CPMR 4126	Awiemfoɔsamena	R, L	**Waist pains,**	Crush (Enema)	100	0.02	0.01
*Dalbergia saxatilis* Hook.f. CPMR 4127	Ahomakyɛm	SB	**Piles**, Ritual	Decoction (Oral/Topical)	66.7	0.02	0.02
*Acacia pycnantha* Benth. CPMR 4128	Akasia	SB	**Wounds**	Grind (Topical).	100	0.02	0.02
*Albizia zygia* (DC.) J.F.Macbr. CPMR 4129	Ɔkorɔ	L	**Blood tonic**	Cooked (Oral)	50	0.03	0.02
			Stomachaches	Decoction (Oral)			
*Abrus precatorius* L. CPMR 4130	Nyame eni	Se	**Epilepsy**	Powder (Oral)	100	0.02	0.02
**Lamiaceae**							
*Hoslundia opposita* Vahl. CPMR 4132	Nunum nini	L, Se	Malaria, **phlegm**	Decoction (Oral)	57.1	0.05	0.03
*Ocimum gratissimum* L. CPMR 4131	Nunum	L, Se	Headache, **Diarrhoea**, Malaria	Decoction (Oral)	38.5	0.37	0.35
			Itchy ear, Phlegm, Convulsion	Squeeze (Topical)			
**Lauraceae**							
*Persea americana* Mill. CPMR 4133	Paya	L	**Fever**	Decoction (Oral)	100	0.06	0.09
**Lecythidaceae**							
*Petersianthus macrocarpus* (P. Beauv.) Liben CPMR 4134	Asia	L	**Constipation**	Grind (Enema)	100	0.02	0.02
**Liliaceae**							
*Allium sativum* L. CPMR 4135	Garlic	Cloves	**Cough**	Raw/paste (Oral)	100	0.04	0.04
**Loganiaceae**							
*Anthocleista nobilis* G. Don CPMR 4066	Owudifo kɛtɛ	R, L	**Piles**, Hernia, Jaundice, Typhoid	Decoction (Oral)	33.3	0.04	0.05
**Loranthaceae**							
*Tapinanthus bangwenis* (Engl.& K. Krause) Danser CPMR 4085	Nkrapan	Wp	*Aseram*, **Fever**	Decoction (Topical)	82.4	0.12	0.13
**Malvaceae**							
*Ceiba pentandra* (L.) Gaertn. CPMR 4069	Onyina	SB, F	**Measles**, Back pain, Sting removal	Grind (Topical)	50	0.03	0.04
*Gossypium hirsutum* L. CPMR 4136	Asaawa	L	Blood Tonic	Decoction drunk	100	0.03	0.03
*Abelmoschus esculentus* Moench. CPMR 4137	Nkruma	F, Se	**Child delivery**, Wounds	Rub (Topical)	80	0.04	0.03
*Sida acuta* Burm. f. CPMR 4138	Tweta	L, R	**Pregnancy care**	Pound (Enema)	57.1	0.05	0.04
		L	Cough, Sore throat	Squeeze (Oral)			
*Cola nitida* (Vent.) Schott & Endl. CPMR 4139	Bese	SB	Ease baby delivery	Grind (Enema)	100	0.02	0.02
**Meliaceae**							
*Khaya senegalensis* (Desv.) A.Juss. CPMR 4141	Mahogany	L, SB	Malaria, Blood pressure	Infusion (Oral)	53.3	0.11	0.12
			**Boils**	Crush (Topical)			
*Trichilia monadelpha* P. Browne. CPMR 4140	Tannuru	L	**Heartburns**, Piles	Pound (Oral)	66.7	0.02	0.01
**Meliaceae**							
*Azadirachta indica* A. Juss. CPMR 4067	Neem tree	L, SB	**Malaria**	Decoction (Oral)	100	0.33	0.25
*Cedrela odorata* L. CPMR 4142	Gyenegyene	SB	**Migraines**	Crush (Oral)	100	0.02	0.02
**Menispermaceae**							
*Sphenocentrum jollyanum* Pierre CPMR 4143	Kramankote	R	Erectile dysfunction	Chew roots and Swallow	100	0.03	0.03
**Moraceae**							
*Ficus exasperata* Vahl. CPMR 4072	Nyankyerɛnee	L,	**Malaria**	Decoction (Oral)	33.3	0.11	0.09
		SB,	Wound, Shingles	Rubbing (Topical)			
		L	Abnormal foetus position	Cooked (Oral)			
*Ficus capensis* Thunb. CPMR 4144	Ɔdoma	L	**Stomachaches**	Decoction (Oral)	100	0.03	0.03
**Moringaceae**							
*Moringa oleifera* Lam CPMR 4145		Se, L	**Ulcer**, Fever	Crush (Oral)	54.5	0.08	0.09
		L	High blood pressure	Squeeze (Oral)			
**Musaceae**							
*Musa paradisiaca* L. CPMR 4146	Brɔdeε	L, R, Fl	**Wounds**	Pound (Topical)	40	0.11	0.1
		L, R	Fever, Headache	Decoction (Oral)			
			Delayed child walk	Grind (Enema)			
**Myristicaceae**							
*Pycnanthus angolensis* (Welw.) Warb. CPMR 4147	Ɔtiɛ	L, SB, L	Pregnancy care	Decoction (Oral)	36.4	0.08	0.11
		SB	Toothache, **Pile**	Crush (Oral)			
**Myrtaceae**							
*Psidium guajava* L. CPMR 4148	Gua	L,	Chicken pox, **Measles**	Rub (topical)	66.7	0.05	0.07
			Candidiasis	Decoction (Oral)			
**Nephrolepidaceae**							
*Nephrolepis biserrata* (Sw.) Schott	Aya	L	**Blood Tonic**	Decoction (Oral)	66.7	0.02	0.02
CPMR 4149			Toothache	Pound (Oral)			
**Pedaliaceae**							
*Sesamum indicum* L. CPMR 4150	Sesame	Se	**Blur eye sight**	Raw (Oral)	100	0.02	0.02
**Phytolaccaceae**							
*Hilleria latifolia* (Lam.) H.Walter CPMR 4151	Anafranaku	L	***Aseram***	Decoction (Bath)	100	0.02	0.03
**Poaceae**							
*Saccharum officinarum* L. CPMR 4152	Ahwedeɛ	L	Malaria	Decoction (Oral)	100	0.03	0.03
*Brachyachne obtusiflora* (Benth.) C.E. Hubb. CPMR 4153	Abirekyire abɔdwesɛ	L,	Fracture, **Delayed child walking**	Paste (Topical/Enema)	75	0.03	0.02
*Zea mays* L. CPMR 4154	Aburoo	R,	Constipation	Paste (Enema)	60	0.04	0.03
		Co	**Anal sore**	Burn (Rectal)			
*Cymbopogon citratus* (DC.) Stapf CPMR 4156	Fever grass	L	**Fever**, Malaria, Typhoid	Decoction (Oral)	61.1	0.13	0.15
Poaceae							
*Bambusa vulgaris* Schrad. ex J.C. Wendl. CPMR 4155	Pampulo	L	Malaria	Decoction (Oral)	100	0.02	0.02
*Eleusine indica* (L.) Gaetn CPMR 4157	Nsensan	Wp	Constipation	Paste (Enema)	100	0.02	0.02
**Rubiaceae**							
*Mussaenda erythrophylla* Schumach. and Thonn. CPMR 4158	Dameramma	L,	**Severe cough,** Ritual	Decoction (Oral)	60	0.02	0.02
*Morinda lucida* Benth. CPMR 4159	Konkrɔma	L	**Malaria**	Decoction (Oral)	100	0.04	0.04
*Psydrax subcordata* (DC.) Bridson var. subcordata CPMR 4160	Ntatiadupon	SB	**Boils**	Crush (Topical)	50	0.04	0.02
			Body pains	Pound (Oral)			
**Rutaceae**							
*Citrus aurantiifolia* (Christm.) Swingle	Ankaa twadeɛ	F, R	**Fever**, Cough, Typhoid	Decoction (Oral)	55.6	0.06	0.06
CPMR 4161			Migraines	Pound (Topical)			
*Zanthoxylum leprieurii* Guill. & Perr	Oyaa	SB,	**Rheumatism**	Pound (Topical)	62.5	0.06	0.06
CPMR 4162			**Stomach ulcer**	Decoction (Oral)			
*Citrus sinensis* (L.) Osbeck CPMR 4163	Ankaa	L	Malaria	Decoction (Oral)	66.7	0.04	0.04
		R/L, Se	**Diarrhoea**	Raw (Oral)			
*Zanthoxylum gilletii* (De Wild.) Waterman CPMR 4164	Okuo	SB	Stomach ulcer	Infusion (Oral)	100	0.03	0.03
**Sapindaceae**							
*Paullinia pinnata* L. CPMR 4079	Toa-ntini	R,	Profuse cough	Raw (Oral)	63.6	0.08	0.12
		R/L	Wounds, snake bite	Paste (Topical)			
		R/L	**Erectile dysfunction**	Alcohol infusion (Oral)			
**Solanaceae**							
*Solanum erianthum* D. Don CPMR 4166	Pepediewuo	L	Piles, **Wounds**	Grind and apply	75	0.02	0.02
*Solanum torvum* Sw. CPMR 4167	Abedru	L/F	**Blood tonic**	Decoction (Oral)	53.3	0.11	0.12
		L	Cough, Typhoid, Headache	Rub (Topical)			
*Solanum lycopersicum* L. CPMR 4168	Ntoose	L	Convulsion	Rub (Topical)	66.7	0.02	0.02
		F	**Measles**	Crush (Topical)			
**Sterculiaceae**							
*Theobroma cacao* L. CPMR 4169	Kookoo	R, L,	Cough	Pound (Oral)	55	0.14	0.14
			**Malaria**	Decoction (Oral)			
**Verbanaceae**							
*Tectona grandis* L.f. CPMR 4086	Teak	L, SB	**Malaria**	Decoction (Oral)	100	0.19	0.16
*Lantana camara* L. CPMR 4075	Shingles dokono	L,	Body pains	Decoction (Oral)	100	0.02	0.02
**Zingiberaceae**							
*Aframomum melegueta* K.Schum. CPMR 4064	Ɛfɔmwisa	Se,	Convulsion	Rub (Topical)	100	0.04	0.04
*Zingiber officinale* Roscoe CPMR 4165	Kakaduro	Rh	**Cough**, Diarrhoea, Wounds	Raw (Oral)	54.5	0.08	0.03

Ethnobotanical indices: UV; use value, FC; frequency of citation, FL; Fidelity Level (The ailments in **bold** were used to calculate the fidelity level). Parts used: R; Roots, L; Leaves, Fl; Flower, SB; Stem bark, Se; Seed, Rh; Rhizome, Wp; Whole plant, Co; Cob, Cv; Cloves, RB; Root bark, F; Fruit, CPMR; Centre for Plant Medicine Research, *Aseram*; Traditional infant disease characterised by green/black visible veins, big head, and lean growth of the baby, *Asabera*; Traditional infant disease which presents as: extreme hot body, pale eyes, and frequent green, foamy bloody stools as significant symptoms.

**Table 3:** Ranked values assigned by each key informant for each of the ten preferred medicinal plants.

Plant Species	Parts Used	Key Ailments Treated	Number of Key Informants (n = 15)															Total Value	Rank
			R1	R2	R3	R4	R5	R6	R7	R8	R9	R10	R11	R12	R13	R14	R15		
*S. alata*	L	Skin diseases	5	7	6	5	4	4	5	4	7	4	6	6	6	4	6	79	8th
*V. amygdalina*	L, R	Malaria, diarrhoea, *asabera*	10	8	7	10	6	7	8	6	8	8	10	8	10	8	8	122	4th
*A. cordifolia*	L, B	Malaria, wounds, piles,	5	5	5	6	4	5	4	6	6	5	6	5	5	4	6	77	9th
*M. charantia*	L	Abdominal pain, fever	7	10	8	6	8	6	5	8	6	8	8	7	6	8	6	107	7th
*S. occidentalis*	L	Stomach aches, phlegm	8	10	8	6	10	10	8	9	8	8	8	10	10	10	8	131	3rd
*T. grandis*	L	Malaria, fever	8	10	8	8	5	6	8	6	9	10	10	6	6	8	8	116	5th
*C. patens*	L, SB	Malaria, fever, piles	7	10	9	8	10	7	10	8	10	8	10	8	10	10	8	133	2nd
*A. boonei*	L, B, R	Measles, piles, chicken pox	9	8	7	6	5	4	6	6	6	5	6	6	8	10	6	98	6th
*A. indica*	L	Malaria	10	10	10	10	10	8	10	10	10	10	8	10	10	10	10	146	1st
*P. urinaria*	Wp	Malaria, typhoid, ulcer	3	5	6	4	3	3	4	6	4	6	5	6	6	6	5	72	10th

**Table 4:** Informant consensus factor (ICF) values for ailment categories of medicinal plants in Ejisu-Juaben Municipality.

No.	Category of Ailment (Specific Conditions)	Number of Taxa (Nt)	Use Citations (Nur)	Informant Consensus Factor (ICF)
1	Pains & Fever: Migraines, headaches, fever, malaria, Abdominal pain, waist and body pains	51	402	0.88
2	Skin diseases: eczema, skin rash, cuts, wounds, shingles, blood clotting, chicken pox, measles, sting removal	29	182	0.85
3	Erectile dysfunction & Impotence: Male sexual vitality, low sperm count	4	17	0.81
4	Gastrointestinal system diseases: Stomach aches, bloating, ulcers, constipation, diarrhoea, Jaundice, Piles, Typhoid, worms, vomiting	50	248	0.80
5	Gynaecological issues and Childcare: Delayed walking, convulsion, abnormal fetus position, *asebera*, *aseram*, family planning, candidiasis	26	98	0.74
6	Respiratory tract infections: cough, sore throat, cold, phlegm, asthma, catarrh	20	73	0.74
7	STDs & Venereal diseases: Gonorrhea,	2	4	0.67
8	Muscular skeletal problems: fractures, rheumatism, joint pains,	5	12	0.64
9	Poisonous animal bites: Snake bite	4	9	0.63
10	Blood problems/Tonic/stimulant: Tonic, low appetite	12	35	0.68
11	Arthritis & inflammation: Swollen body parts, hernia, boils, gum diseases, toothache	10	22	0.57
12	Eye and ear infections: blur eyesight, itching ear,	6	10	0.44
13	Neurological & nervous system disorders: Convulsions, epilepsy	4	6	0.40
14	Cardiovascular diseases: heart burns, high blood pressure, stroke	8	12	0.36
15	Other ailments: bedwetting, rituals, To stop alcoholism,	3	4	0.33
	Total	234 *	1134	

* A Taxa may be reported in more than one ailment category.

**Table 5:** Priority ranking of factors perceived by key informants as threats to medicinal plants in the Ejisu-Juaben Municipality

Major Threat	Number of Key Informants (n = 15)															Total Value	Rank
	R1	R2	R3	R4	R5	R6	R7	R8	R9	R10	R11	R12	R13	R14	R15		
Agricultural activities	4	5	4	4	5	4	5	4	4	5	4	4	5	4	4	65	1st
Fodder/Overgrazing	1	0	0	1	0	2	1	0	0	1	1	0	1	2	1	11	7th
Building and construction	2	1	1	2	1	1	2	1	1	2	2	1	1	2	1	21	6th
Bush fires	3	2	3	3	2	3	3	1	2	3	2	2	2	3	2	36	4th
Drought	3	3	2	4	3	3	2	3	3	3	4	2	2	4	3	44	2nd
Floods	0	1	0	1	0	1	0	0	1	0	1	1	0	0	1	7	8th
Firewood/Fuel	2	3	3	2	2	3	3	2	2	2	1	2	2	3	2	34	5th
Excessive harvesting	3	3	2	2	3	2	2	3	2	3	3	2	3	2	3	38	3rd

Key: Values 1–5 were given: 1 is the least destructive threat, and 5 is the most destructive threat.

## Supplementary Materials

The following are available online at http://www.mdpi.com/2305-6320/6/1/1/s1, File S1: Sample of the questionnaire for interviews, File S2: Medicinal plants, their habit, parts used, ailments treated, cultivation status, method of preparation and administration in the Ejisu-Juaben Municipality.
