# Supplementary material for: Medicinal Plants Used in the Ejisu-Juaben Municipality, Southern Ghana: An Ethnobotanical Study
Source: Medicines (Basel). 2018 Dec 20;6(1):1. doi: 10.3390/medicines6010001 (PMC6473417; doi:10.3390/medicines6010001)
Supplement: Supplementary file 1 [file medicines-06-00001-s001.zip › medicines-401634 supplementary proof done/File S1-sample of questionnaire.pdf]

Appendix A: sample of questionnaire for interviews

This study is being carried out by Kwame Sarpong Appiah, a graduate student at the Tokyo University of Agriculture and Technology, Japan on "Medicinal plants used in the Ejisu-Juaben Municipality, Southern Ghana: An ethnobotanical study". All information gathered will be treated with much confidentiality and would solely be used for academic purposes. Your support and contribution would be very much appreciated.

Name of Enumerator/Interviewer…………………Date........…Questionnaire number...
Locality/Town/Village/Community……………… Contact…………………………………..

PERSONAL INFORMATION (Part I)

1. Name of respondent………………………………………………

2. Phone number……………………..

3. Sex: Male [ ] Female [ ]

4. Age: ...

5. Marital status: Married [ ] Widow/Widower [ ] Single parent [ ] Divorced/Separated [ ] Other (please specify)……..…

6. Religious affiliation: Christian [ ] Muslim [ ] Traditionalist [ ] No religious affiliation [ ] Other (please specify)…….

7. Level of education: None [ ] Basic (Primary/JHS/Middle) [ ] Secondary [ ] Tertiary (Training College/Polytechnic/University) Other (please specify…………

8. Which ethnic group/tribe do you belong to? ....

9. Main occupation …………………………………………………..

10. What is your reason for using medicinal plants for health issues? Part of culture [ ] Freely available [ ] Western medicine cannot cure some diseases [ ] Conventional medicine results in too many side effects [ ] Others (please specify)…………...………………………………

11. Where do you obtain the medicinal plants you use? Self-collection [ ] Herbal market [ ] Herbal clinic [ ] Traditional herbalist [ ] others (specify)………………………

12. How do you obtain information regarding the kinds of diseases the medicinal plants can cure? Experience of others [ ] Media [ ] Books [ ] Herbalist [ ] Spiritual intuition Others (please specify)……………

13. Do you get the treatment you require from medicinal plants?
Yes [ ] No [ ] Somehow

14. For how long have you been using herbal medicine? ....

Medicinal plant and diseases Information (Part II)

15. Name of the medicinal plant ……………………

16. The local name(s) of medicinal plants……………………..

17. Name of ailment treated (also local name)……………….

18. Collection number for plant……………………

19. Plant parts used: Leaves [ ] Root/root bark [ ] Stem bark [ ] Whole plant [ ] Flower [ ] Fruit [ ] Seed [ ] others (please specify)…………………………

20. Condition of plant material used: Fresh [ ] Dried [ ] Burnt [ ]

21. Where do you collect the medicinal plant? Everywhere [ ] In the bush [ ] Near home [ ] Cropland [ ]

Methods of preparation and mode of administration of herbals remedies

22. How do you prepare the herbal remedy?

Decoction [ ] Infusion [ ] Grinding [ ] Crushing [ ] Eaten raw [ ] Others (please specify)……………………

23. What other additives do you add to the herbal remedy:…………………….

24. How do you administer the herbal remedy? Drinking [ ] Bathing [ ] Enema [ ] Body massage [ ] Inhalation [ ] Steam bath [ ] Eye drop [ ] Eardrop [ ] others (please specify)…………………………………

25. Are there any side effects related to this herbal remedy………………..

Ranking Part III (for only key informers)

26. What are the threats to the availability of medicinal plants?

**Threat**	**Score value**
1.	
2.	
3.	
4.	
5.	
6.	
7.	
8.	
9.	

Key: Values 1–5 were given: 1 is the least destructive threat and 5 is the most destructive threat.

27. Which plants are the most preferred based on availability and effective to treating diseases?

**Medicinal plant**	**Plant part used**	**Key ailment treated**	**Score value**
1.			

2.			
3.			
4.			
5.			
6.			
7.			
8.			
9.			
10.			

10: Highest scores (readily available and very effective)
1: Lowest score (rarely available and less effective)

Thank you for your cooperation.
